# Intrauterine device (IUD) embedded in bladder wall with calculus formation treated with cystoscopy: a case report

**DOI:** 10.1093/omcr/omae073

**Published:** 2024-07-21

**Authors:** Van Trung Hoang, Ny Ny Thi Le, The Huan Hoang, Vichit Chansomphou

**Affiliations:** Department of Radiology, Thien Hanh Hospital, 17 Nguyen Chi Thanh Street, Buon Ma Thuot 630000, Vietnam; Department of Radiology, Nguyen Tri Phuong Hospital, 468 Nguyen Trai Street, Ward 8, District 5, Ho Chi Minh City 70000, Vietnam; Department of Radiology, Thien Hanh Hospital, 17 Nguyen Chi Thanh Street, Buon Ma Thuot 630000, Vietnam; Radiology Department, Savannakhet Medical-Diagnostic Center, 266/5 Chaimeuang Phetsarat Street, Kaysone Phomvihane 13000, Lao People’s Democratic Republic

**Keywords:** bladder-embedded intrauterine device, bladder stone, cystoscopy, intrauterine device migrations, IUD migration

## Abstract

In the realm of unusual gynecological complications, the displacement of an intrauterine device (IUD) into the bladder, resulting in stone formation, stands out as an exceptionally rare and perplexing condition. Such occurrences challenge diagnostic and therapeutic protocols, often leading to unique case studies that expand our understanding of IUD-related complications. We present an interesting case of a 50-year-old woman with a stone-forming ectopic IUD in the bladder diagnosed with imaging modalities and treated with cystoscopy, with a subsequent resolution of symptoms. This case underscores the importance of considering ectopic IUD placement in the differential diagnosis of patients presenting with urinary symptoms and a history of IUD use. Moreover, it emphasizes the role of imaging in the accurate diagnosis of such cases and highlights cystoscopy as an effective treatment modality for the removal of IUD and stones.

## Introduction

Worldwide, the intrauterine device (IUD) is currently the most popular means of reversible birth control in the world with 160 million users [1]. As with any medical device, its administration can lead to complications such as perforation and extrauterine migration to adjacent organs. The rate of IUD migration is very low, ranging from 0.1% to 0.9% [2, 3]. The most common sites for IUD migration are the omentum, rectum, sigmoid colon, peritoneum, and bladder [4]. The incidence of an IUD translocation into the bladder wall is rare, with only isolated cases reported [3, 5, 6]. Hence, awareness of such situations is crucial.

## Case presentation

**Figure 1:**
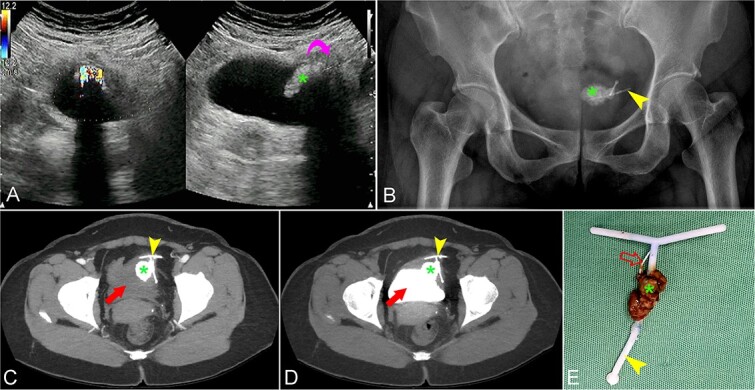
(**A**) Transabdominal ultrasound images showed a stone (asterisk) attached to the bladder wall with a thickened adjacent bladder wall and loss of layered structure (curved arrow). The initial potential diagnosis was a calcified tumor or a stone attachment to the bladder wall or a stone-forming foreign body. Initially, the IUD could not be seen on ultrasound. (**B**) X-ray showed a T-shaped IUD (arrowhead) attached to a calcified mass (asterisk). (**C**) CT unenhanced phase and (**D**) contrast-enhanced phase with the delayed images showed the IUD (arrowheads) penetrating the bladder wall into the bladder lumen (arrows) and stone formation (asterisks). (**E**) The specimen image showed a T-shaped IUD (arrowhead) with a partial cord attachment (open arrow) and an attached calculus (asterisk). Note that part of the IUD cord was still in the bladder wall and could not be removed and part of the stone mass has been crushed.

A 50-year-old woman presented to the hospital with lower urinary tract symptoms (LUTS), including suprapubic discomfort, frequency, and a feeling of incomplete voiding for the past 2 months. The patient had a history of three vaginal deliveries, no abortions, and no C-sections. After giving birth to her last child about 17 years ago, her period returned 3 months later and she had an IUD inserted at a lower-level hospital in the 4th month after giving birth. After that, she had no discomfort and no follow-up. Her physical examination was normal. Complete blood count showed an elevated white blood cell count of about 12 300/ml. Kidney function showed normal. Urinalysis showed WBC 3+, RBC 1+, and epidermal cells 1+. Urine culture results were positive for *Escherichia coli*. A transabdominal ultrasound showed that a calcified structure attached to the left anterior wall of the bladder was approximately 23 × 30 mm in dimension, the adjacent bladder wall thickened, and the layer structure was unknown (Fig. 1A). X-ray and computed tomography (CT) images showed that a T-shaped IUD attached to the bladder wall and intravesical stone formation. Most of the IUD was located in the bladder wall, one end of the T-arm of the IUD was in the peritoneum, a middle part of the T-leg of the IUD was exposed into the bladder lumen and formed stones (Fig. 1B–D). The uterus appeared normal on ultrasound and CT scan with an anteroposterior diameter of approximately 25 mm. The patient was treated for urinary infection with quinolone antibiotics according to the results of the antibiogram, with ciprofloxacin at a dose of 500 mg orally twice a day for 7 days. At the same time, she underwent cystoscopy with spinal anesthesia. The stone attached to the bladder wall was dispersed by laser. After the stone was partially dissolved, the rest of the stone and the IUD that penetrated the bladder wall fell out by itself into the bladder lumen. Crushed the gravel and pumped out all the gravel, then removed the IUD (Fig. 1E). A portion of the IUD’s cord remained in the bladder wall, which could not be withdrawn, decided to leave the cord in the bladder wall. Observing no bleeding complications, no bladder fistula, and no bladder wall defect, it was decided not to have laparoscopy to repair the bladder wall. Urethral and bladder Foley catheter was kept postoperatively for 5 days and was removed after an ultrasound examination showed no bladder stones and no complications. Micturating cystogram was recommended but the patient refused to have it performed. However, during this period, hematuria was not observed. The patient was discharged after 6 days of treatment without complications. Followed up for more than 1 year, the patient had no symptoms of urinary tract abnormalities and subsequent ultrasounds only noted mild thickening of the bladder wall in the left anterior position.

## Discussion

IUD migration is an uncommon condition. The exact mechanism of the IUD migration is unclear. There is a consensus that the common mechanism is that the device is forced into and through the uterine wall upon insertion. Perforation is more likely to occur in the early stages or soon after IUD insertion [3, 6, 7]. Factors influencing uterine perforation include low experience of the placing provider, postpartum placement (< 6 months from delivery), and low parity [4, 5, 8]. During the puerperium and lactation period, the uterine wall is thin and soft, and the possibility of IUD migration is the greatest. Uterine perforation can be partial or complete, depending on whether the device completely penetrated penetrates the uterine wall [2, 5, 8]. In our case, we could not determine the exact cause of IUD migration in the uterus. Clinical symptoms of ectopic IUD vary from patient to patient and depending on the location of the IUD, symptoms may vary including asymptomatic, colporrhagia, hypogastralgia, abdominal pain, and lower urinary tract syndrome (increased urinary frequency, urinary urgency, dysuria, and hematuria) [3, 4, 9]. Imaging methods (including ultrasound, computed tomography, and plain X-ray) and cystoscopy provide strong diagnostic support and play an important role in the selection of treatment methods and surgical approaches [4, 5, 9]. The treatment options are highly associated with the location of the migrated IUD. It is also related to the shape of the IUD, the patient’s conditions, and hospital equipment [3, 5]. Kiilholma reported a case in which the IUD partially penetrated the bladder but the string remained in the cervix and the IUD was successfully removed through the vagina by string extraction [10]. If the IUD with stone is located mostly in the bladder lumen, cystoscopy is the optimal method to remove the IUD and stone [6, 8]. If the IUD is located mostly within the wall or outside the bladder lumen, surgery is recommended [3, 5, 7, 9]. Laparoscopy is preferred over open surgery, and spinal anesthesia is preferred over general anesthesia [2, 11, 12]. In cases of failed cystoscopy, surgery should be considered. In the literature, most of the reported cases were treated with laparoscopic and open surgical procedures [2–4, 7, 8, 11, 13, 14]. Only a few rare cases reported in which cystoscopy was performed to remove the IUD [5, 6]. In our case, the IUD had become totally separated from the uterus and was embedded in the urinary bladder wall, which raised the possibility of its removal with cystoscopy alone. However, a portion of the IUD’s cord remained in the bladder wall, which could not be withdrawn, decided to leave the cord in the bladder wall. Followed up for more than 1 year, the patient had no symptoms of urinary tract abnormalities and subsequent ultrasounds only noted mild thickening of the bladder wall in the left anterior position. Potential complications due to a residual IUD in the bladder wall can range from asymptomatic to variable symptoms such as fever, malaise, nausea, abdominal pain, dysuria, urinary urgency, hematuria, cloudy urine, inflammatory reaction with fibrosis and granulation tissue formation, abscess, bladder perforation, cellular dysplasia and possible tumor formation, or possible adhesions of surrounding bowel loops [3, 4, 7, 8, 12, 14]. Routine examination with urinalysis and abdominal ultrasound can help detect these complications [6, 9, 13, 14].

## Conclusion

Chronic pelvic pain and irritative voiding symptoms with a history of an unretrieved IUD must be carefully researched for possible perforation of the uterus and intravesical IUD. When migration of the intrauterine contraception device is present, removal is mandatory due to its potential complications. Cystoscopic translocated IUD removal can be considered an effective and safe minimally invasive approach to managing an ectopic IUD in the urinary bladder. Through this report, we aim to increase awareness among healthcare professionals about this potential complication of IUD use, thereby improving patient outcomes through timely diagnosis and treatment.
